# Dual Glucagon-Like Peptide-1 (GLP-1) and Glucose-Dependent Insulinotropic Polypeptide (GIP) Receptor Agonist-Associated Thyroiditis: A Case Report of Thyroid Dysfunction Following Tirzepatide Use

**DOI:** 10.7759/cureus.85123

**Published:** 2025-05-31

**Authors:** Sara Humaida, Kamar Manzalji, Naheel Seyam, Lolwa Al-Masalmani

**Affiliations:** 1 Family Medicine, Primary Health Care Corporation, Doha, QAT

**Keywords:** drug-induced thyroiditis, glp-1 receptor agonist, hypothyroidism, thyroid dysfunction, tirzepatide

## Abstract

Drug-induced thyroiditis is an uncommon but clinically important condition. As dual glucagon-like peptide-1 (GLP-1) and glucose-dependent insulinotropic polypeptide (GIP) receptor agonists like tirzepatide are increasingly used for weight management and blood sugar control, there is growing interest in understanding their potential thyroid-related effects.

We report a 32-year-old woman with no personal or family history of thyroid disease who developed painless biphasic thyroiditis, initial thyrotoxicosis followed by transient hypothyroidism, after two months of tirzepatide therapy. Thyroid autoantibodies were negative, and ultrasound showed heterogeneous echotexture with increased vascularity, consistent with thyroiditis. Infectious, autoimmune, postpartum, and infiltrative causes were excluded based on clinical history, laboratory findings, and imaging. Thyroid function normalized two months after discontinuing tirzepatide without the need for treatment.

This case highlights a possible association between tirzepatide and drug-induced painless thyroiditis. It adds to the limited literature and emphasizes the need for clinician awareness of this possible adverse effect.

## Introduction

Dual glucagon-like peptide-1 (GLP-1) and glucose-dependent insulinotropic polypeptide (GIP) receptor agonists, including tirzepatide, are increasingly prescribed for type 2 diabetes and obesity due to their efficacy in glycemic control and weight reduction. While gastrointestinal side effects are the most commonly reported, thyroid safety has garnered attention primarily because of preclinical studies in rodents showing C-cell hyperplasia and a possible risk of medullary thyroid carcinoma [1].

Thyroid-related adverse events, including hypothyroidism and thyroiditis, have been reported in pharmacovigilance databases and systematic reviews. A recent meta-analysis of randomized controlled trials reported a 28% increased risk of overall thyroid disorders with GLP-1 receptor agonist use, though the association with hypothyroidism or thyroiditis specifically was not statistically significant [2]. Additionally, pharmacovigilance analyses using the FDA Adverse Event Reporting System (FAERS) noted an increased number of thyroid-related reports, including hyperplasia and neoplasms, associated with GLP-1 receptor agonists. While these reports are subject to limitations such as self-reporting bias and lack of confirmed causality, they may provide important early signals that warrant further clinical investigation [3].

It is important to acknowledge that subacute (viral) thyroiditis remains a far more common etiology of thyroid inflammation and dysfunction. Drug-induced thyroiditis, while relatively rare, is a recognized phenomenon associated with certain medications, including interferons, amiodarone, lithium, and tyrosine kinase inhibitors [4,5]. Proposed mechanisms include immune dysregulation and direct cytotoxic effects on thyroid follicular cells.

In this report, we describe a case of thyroiditis that developed shortly after the initiation of tirzepatide in a previously euthyroid individual. While a definitive causal link cannot be established, the temporal relationship, exclusion of common viral symptoms, and recovery after drug discontinuation raise the possibility of a drug-related adverse event. This case highlights the need for heightened awareness and further investigation into the thyroid safety profile of GLP-1/GIP receptor agonists.

## Case presentation

A 32-year-old woman with no personal or family history of thyroid disease presented to the primary care clinic with a one-week history of gradually progressive palpitations, heat intolerance, and fatigue. She denied any recent viral illnesses, sore throat, fever, or neck pain. There were no similar episodes in the past, and she was not taking any prescribed medications, over-the-counter supplements, or herbal products. She is a mother of three, with her youngest child being four years old, and denied any recent pregnancy or delivery within the last 12 months, making postpartum thyroiditis unlikely.

Her medical history was unremarkable for autoimmune or endocrine disorders, and she was a non-smoker with no history of radiation exposure to the head or neck.

Three months prior to presentation, she had initiated tirzepatide (branded FDA-approved formulation) for weight management under medical supervision. The treatment had initially been well tolerated, and baseline laboratory work-up, including thyroid function tests (TFTs), was within normal limits. Approximately one month after starting tirzepatide, the patient began to experience symptoms consistent with thyroid dysfunction.

On examination, the patient was alert and hemodynamically stable. Neck examination revealed a mildly enlarged, non-tender thyroid gland without palpable nodules or cervical lymphadenopathy, findings consistent with thyroiditis. There were no signs of orbitopathy or pretibial myxedema. Cardiovascular and other systemic examinations were unremarkable.

Initial laboratory investigations demonstrated a suppressed thyroid-stimulating hormone (TSH) of 0.01 mIU/L and an elevated free thyroxine (free T4) of 2.37 ng/dL, consistent with thyrotoxicosis. Inflammatory markers, including erythrocyte sedimentation rate (ESR) 30 mm/hr and C-reactive protein (CRP) 22 mg/L, were elevated. Thyroid autoantibodies, including anti-thyroid peroxidase (TPO), thyroglobulin antibodies, and TSH receptor antibodies, were all negative. Relevant laboratory results over the course of illness are summarized in Table 1.

**Table 1:** Laboratory findings of the patient with reference ranges TSH: thyroid-stimulating hormone; free T4: free thyroxine; anti-TPO: anti-thyroid peroxidase; ESR: erythrocyte sedimentation rate; CRP: C-reactive protein

Test	Initial result	Six-week result	Two-month post-discontinuation	Normal range
TSH	0.01 mIU/L	15.2 mIU/L	2.1 mIU/L	0.4-4.5 mIU/L
Free T4	2.37 ng/dL	0.54 ng/dL	1.05 ng/dL	0.70-1.48 ng/dL
Anti-TPO antibodies	Negative	N/A	N/A	Negative
Thyroglobulin antibodies	Negative	N/A	N/A	Negative
TSH receptor antibodies	Negative	N/A	N/A	Negative
ESR	30 mm/hr	N/A	N/A	0-20 mm/hr
CRP	22 mg/L	N/A	N/A	<5 mg/L

**Figure 1:**
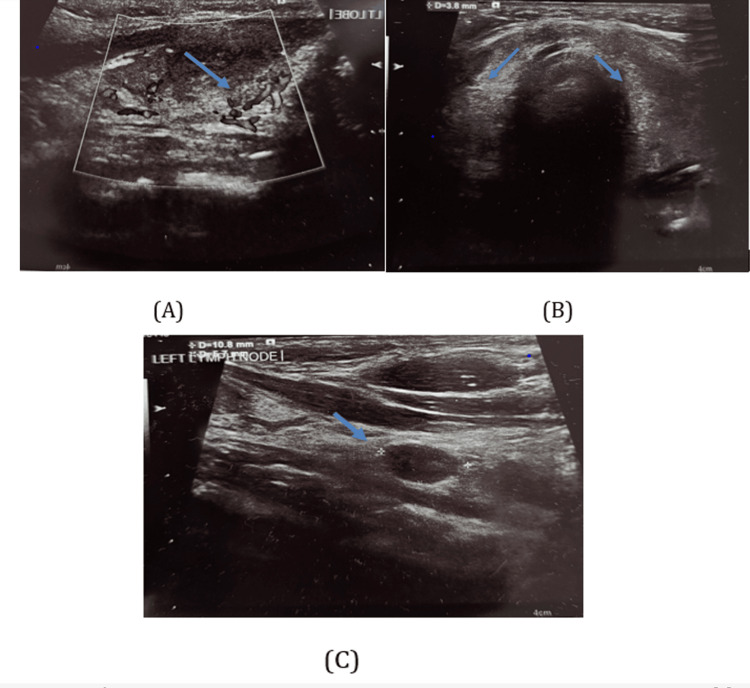
Thyroid ultrasound findings consistent with thyroiditis. (A) Increased vascularity of the thyroid gland. (B) Heterogeneous echotexture with bilateral bulky thyroid lobes and isthmus. (C) Multiple hypoechoic pre- and paratracheal lymph nodes, suggestive of reactive or inflammatory changes

A thyroid ultrasound revealed increased vascularity of the thyroid gland (Figure 1A) and a heterogeneous echotexture with bilateral bulky lobes and isthmus (Figure 1B). Several hypoechoic pre- and paratracheal lymph nodes with loss of the fatty hilum were also identified (Figure 1C), findings suggestive of reactive or inflammatory changes.

Over the following weeks, the patient's hyperthyroid symptoms (palpitations, heat intolerance, and fatigue) began to improve without the need for anti-thyroid therapy. However, at six weeks, she developed new symptoms of fatigue and dry skin. Repeat thyroid function tests revealed elevated TSH (15.2 mIU/L) and low free T4 (0.54 ng/dL), consistent with the hypothyroid phase of painless thyroiditis. Given the temporal relationship with the initiation of tirzepatide and the absence of other triggers (e.g., infection, postpartum state, or medication use), a diagnosis of drug-induced painless thyroiditis was made. Tirzepatide was discontinued at this point. Two months after discontinuation, her thyroid function normalized (TSH: 2.1 mIU/L; free T4: 1.05 ng/dL), and she remained clinically and biochemically euthyroid at three-month follow-up without requiring any thyroid hormone replacement.

## Discussion

Thyroiditis refers to a group of inflammatory thyroid disorders that often present with a biphasic clinical course, beginning with transient thyrotoxicosis due to the release of preformed thyroid hormones, followed by a hypothyroid phase as hormone stores are depleted [4]. Silent or painless thyroiditis is a subtype of subacute thyroiditis typically characterized by a lack of thyroid pain, negative thyroid autoantibodies, and spontaneous resolution. In this case, the clinical course, seronegative autoimmune profile, and ultrasound findings are consistent with painless (silent) thyroiditis.

Drug-induced thyroiditis is a recognized but relatively rare condition, often associated with medications such as interferons, amiodarone, lithium, and tyrosine kinase inhibitors [2,3]. While the mechanism in these cases may involve immune modulation or cytotoxic effects on thyroid follicular cells, GLP-1 receptor agonists have primarily been linked to C-cell effects in animal models. However, emerging data and case reports suggest possible broader, underrecognized impacts on thyroid function, potentially through immune or inflammatory pathways [1].

In this case, the patient developed thyroid dysfunction shortly after the initiation of tirzepatide, with the normalization of thyroid function following the discontinuation of tirzepatide. Although the overall course was consistent with painless, transient thyroiditis, which is often self-limiting, the temporal relationship with tirzepatide use and the absence of other plausible etiologies support a potential drug-related effect. Other common causes of thyroiditis were systematically excluded: the patient had no recent viral illness, was not in the postpartum period, had no personal or family history of autoimmune thyroid disease, and had not been exposed to other known thyroid-disrupting medications. Thyroid autoantibodies (including anti-TPO and anti-thyroglobulin) were negative, and inflammatory markers were within normal limits.

While causality cannot be definitively established based on a single case, the combination of clinical findings, laboratory results, and symptom resolution after drug cessation suggests a possible association with tirzepatide. We acknowledge this is a hypothesis-generating observation and should be interpreted with caution. Further clinical and mechanistic studies are warranted to explore the potential thyroid-related effects of dual incretin receptor agonists, including possible immune-mediated mechanisms.

This is further supported by a Naranjo adverse drug reaction score of 6, indicating a probable drug-induced reaction. Although the Naranjo scale was developed in 1981, it continues to be widely utilized and validated as a reliable tool for assessing causality in adverse drug reactions across various clinical settings. Its use in this case provides an objective framework to support the association between tirzepatide and thyroid dysfunction [6]. The detailed scoring using the Naranjo algorithm is presented in Table 2.

**Table 2:** Assessment of causality using the Naranjo scale A score of 5-8 suggests a probable adverse drug reaction.

Question	Yes	No	Don't know	Score
1. Are there previous conclusive reports on this reaction?		✔		0
2. Did the adverse event appear after the suspected drug was given?	✔			+2
3. Did the adverse reaction improve when the drug was discontinued?	✔			+1
4. Did the adverse reaction reappear when the drug was re-administered?			✔	0
5. Are there alternative causes that could have caused the reaction?	✔			+2
6. Did the reaction reappear when a placebo was given?		✔		0
7. Was the drug detected in blood or other fluids in toxic concentrations?		✔		0
8. Was the reaction more severe when the dose was increased?		✔		0
9. Did the patient have a similar reaction to a similar drug previously?		✔		0
10. Was the adverse event confirmed by any objective evidence?	✔			+1
Total score				6

Emerging literature suggests a potential association between GLP-1 receptor agonists and thyroid disorders. For example, a meta-analysis of 45 randomized controlled trials observed a trend toward increased risk of thyroid dysfunction in patients treated with GLP-1 receptor agonists, although the findings did not reach statistical significance. Therefore, while these data indicate a possible correlation, they do not confirm a causal relationship, and further studies are needed to clarify this potential link [2].

Preclinical studies have further complicated the safety narrative surrounding GLP-1 receptor agonists. Rodent models have demonstrated C-cell hyperplasia and medullary thyroid carcinoma in response to chronic GLP-1 stimulation [1]. This appears to be species-specific due to the higher density of GLP-1 receptors in rodent thyroid C cells compared to humans [1]. Nevertheless, these findings prompted black box warnings on certain GLP-1 receptor agonists and have fueled ongoing post-marketing safety monitoring. The potential for GLP-1 receptor agonists to cause inflammatory thyroid disorders such as thyroiditis is less well characterized and has not been a focus of early trials or safety assessments.

Mechanistically, the thyroidal effects of GLP-1 receptor agonists are not fully understood. GLP-1 receptors have been identified in various extra-pancreatic tissues, including the hypothalamus, heart, and gastrointestinal tract [7,8], but their presence in human thyroid tissue remains controversial. It has been hypothesized that GLP-1 receptor agonists may indirectly affect thyroid function via immune modulation, cytokine release, or alteration of TSH secretion through central neuroendocrine pathways [7]. Additionally, weight loss-related hormonal changes, including reductions in leptin and alterations in T3/T4 metabolism, may influence thyroid axis stability [7,8].

Recently, isolated case reports have also described thyroid dysfunction associated with other GLP-1 receptor agonists. For example, a case of semaglutide-induced subclinical hypothyroidism was reported, where a patient developed elevated TSH with normal free thyroid hormone levels following semaglutide use [9], which subsequently improved upon discontinuation. Although the mechanism remains unclear, this case supports the notion that GLP-1 receptor agonists may have broader thyroidal effects beyond neoplasia, possibly through subtle immune or endocrine modulation.

This is considered the first case that illustrates a possible association between tirzepatide use and the development of silent thyroiditis. While the patient's clinical course was consistent with typical transient thyroiditis, several common causes were systematically excluded, including recent viral illness, autoimmune thyroid disease, postpartum status, and exposure to other medications known to affect thyroid function. The temporal relationship between tirzepatide initiation and symptom onset, followed by normalization of thyroid function after tirzepatide discontinuation, raises the possibility of a drug-related effect. However, this observation does not establish causality, and confounding factors cannot be ruled out.

As dual GLP-1/GIP receptor agonists continue to be prescribed widely for type 2 diabetes and obesity, vigilance regarding their potential thyroid effects is crucial. Though routine thyroid monitoring for all patients on GLP-1 receptor agonists is not currently recommended, thyroid function testing may be warranted in selected patients who develop suggestive symptoms during therapy. Further clinical studies are needed to define the true incidence, risk factors, and mechanisms of GLP-1 receptor agonist-associated thyroid dysfunction, including both neoplastic and inflammatory pathologies.

## Conclusions

This case highlights a possible association between tirzepatide, a dual GLP-1/GIP receptor agonist, and the development of painless thyroiditis characterized by a transient biphasic course of thyrotoxicosis followed by hypothyroidism. Importantly, thyroid function normalized after the discontinuation of tirzepatide, indicating a possible temporal relationship. As GLP-1 receptor agonists are increasingly used for weight management and glycemic control, clinicians may consider thyroid dysfunction in the differential diagnosis when relevant symptoms arise during therapy, even in patients without traditional risk factors. Further pharmacovigilance studies and mechanistic research are warranted to better understand this potential adverse effect and clarify causality.
